# *LMNA* Mutations G232E and R482L Cause Dysregulation of Skeletal Muscle Differentiation, Bioenergetics, and Metabolic Gene Expression Profile

**DOI:** 10.3390/genes11091057

**Published:** 2020-09-07

**Authors:** Elena V. Ignatieva, Oksana A. Ivanova, Margarita Y. Komarova, Natalia V. Khromova, Dmitrii E. Polev, Anna A. Kostareva, Alexey Sergushichev, Renata I. Dmitrieva

**Affiliations:** 1 National Almazov Medical Research Centre, Institute of Molecular Biology and Genetics, 197341 Saint-Petersburg, Russia; lefutr@mail.ru (E.V.I.); astroksana@gmail.com (O.A.I.); komarovamy96@yandex.ru (M.Y.K.); khromova1610@yandex.ru (N.V.K.); akostareva@hotmail.com (A.A.K.); 2 ITMO University, Information Technologies and Programming Faculty, International Laboratory of Bioinformatics and Genomics, 197101 St. Petersburg, Russia; alsergbox@gmail.com; 3 Research Resource Center “Biobank”, St Petersburg State University, 199034 Saint-Petersburg, Russia; brantoza@gmail.com

**Keywords:** laminopathies, muscle dystrophies, *LMNA* G232E/R482L mutations, myogenesis, transcriptome sequencing, bioenergetics, mitochondrial respiration, glycolysis

## Abstract

Laminopathies are a family of monogenic multi-system diseases resulting from mutations in the *LMNA* gene which include a wide range of neuromuscular disorders. Although lamins are expressed in most types of differentiated cells, *LMNA* mutations selectively affect only specific tissues by mechanisms that remain largely unknown. We have employed the combination of functional in vitro experiments and transcriptome analysis in order to determine how two *LMNA* mutations associated with different phenotypes affect skeletal muscle development and metabolism. We used a muscle differentiation model based on C2C12 mouse myoblasts genetically modified with lentivirus constructs bearing wild-type human *LMNA* (WT-*LMNA*) or R482L-*LMNA*/G232E-*LMNA* mutations, linked to familial partial lipodystrophy of the Dunnigan type and muscular dystrophy phenotype accordingly. We have shown that both G232E/R482L-*LMNA* mutations cause dysregulation in coordination of pathways that control cell cycle dynamics and muscle differentiation. We have also found that R482/G232E-*LMNA* mutations induce mitochondrial uncoupling and a decrease in glycolytic activity in differentiated myotubes. Both types of alterations may contribute to mutation-induced muscle tissue pathology.

## 1. Introduction

Laminopathies are the family of monogenic multi-system disorders that result from mutations in the *LMNA* gene, which encodes for nuclear lamins A and C. In the course of two decades, over 400 mutations in *LMNA* have been linked to various forms of laminopathies, including muscle dystrophies, cardiomyopathies, lipodystrophies, and neuropathies [1,2,3,4,5]. Although lamins are expressed in most differentiated tissues, particular *LMNA* mutations selectively affect only specific tissues by mechanisms that remain largely unknown. Genotype–phenotype association studies have raised the hypotheses that different laminopathies selectively affect specific tissues through the ability of nuclear lamina to influence the chromatin status under differentiation and mechanical stress in a mutation-specific manner [6,7,8,9,10].) Namely, if the repairing/regenerating myofibers express the mutant *LMNA*, they demonstrate the compromised and ineffective regeneration capacity and satellite cells’ replicative senescence resulting in higher susceptibility to mechanical damage [11,12,13]. Additionally, *LMNA* mutations may impact the function of the nuclear envelope as a signaling platform, affecting the dynamics of epigenomic perturbations and gene expression in regenerating skeletal muscle [14,15].

Metabolic dysfunction often accompanies muscular dystrophies. Metabolic dysfunction often accompanies muscular dystrophies. Duchenne Muscular Dystrophy (DMD) is characterized by global metabolic alterations: myocytes of DMD patients and mdx mice (mdx mice possess a spontaneous point mutation in exon 23 of dystrophin that prevents expression of full-length dystrophin; the model is used for studying DMD) exhibit reduced oxygen consumption, spare capacity, and mitochondrial complex I activity [16]. The impairment of mitochondrial oxidative phosphorylation, consistent with a reduction in expression of genes coding for mitochondrial enzymes, was described in DMD, muscular dystrophy associated with α-sarcoglycan deficiency and dystrophin-deficient mdx mice [17,18]. Primary cells form patients with lamin-associated lipodystrophy and corresponding animal models also reveal metabolic alterations, providing evidence for the role of lamin A/C in regulating cell metabolism [19]. Even though plenty of *LMNA* mutations are linked to muscle dystrophies [20], data about skeletal muscle bioenergetics in laminopathies are very limited. Muscular metabolic defects in fatty acid and glucose metabolism have been shown for FPLD (familial partial lipodystrophy) and LGMD1B (Limb-girdle muscular dystrophy) patients [21], and some evidence of mitochondrial dysfunction were shown in human fibroblasts expressing *LMNA* mutations [22,23].

In order to determine how *LMNA* mutations associated with different clinical phenotypes affect the molecular mechanisms that control skeletal muscle differentiation and metabolism we investigated the mutation-specific transcriptome and bioenergetic changes in C2C12 myobasts harboring R482L and G232E *LMNA* mutations. These two mutations are linked to different types of laminopathies. The first one, R482L, has been reported in patients with familial partial lipodystrophy of the Dunnigan type (FPLD2; OMIM no. 151660) which primarily affect adipose tissue but, in many cases, is accompanied by skeletal muscle involvement [24,25,26,27]. The sporadic mutation G232E in *LMNA* was described by Bonne and co-authors in a patient with early onset and severe muscular dystrophy with signs of LGMD and EDMD (Emery-Dreifuss muscular dystrophy) phenotypes and no cardiac involvement [1].

In our recent reports we described the alterations in the dynamics of C2C12 mouse myoblast myogenic differentiation and myofiber morphology caused by R482L/G232E-*LMNA* mutations [25,28]. In the present work we provide the insight into the molecular mechanisms behind these alterations. Using functional studies, transcriptome analysis, and mitochondrial respiration screens we demonstrate that both mutations lead to the dysregulation of the muscle differentiation/reparation program and the alterations of muscle cell bioenergetics in a mutation-specific manner.

## 2. Materials and Methods

### 2.1. Cell Culture and Myogenic Differentiation

C2C12 mouse myoblasts cell line was pursued from ATCC (ATCC CRL-1772). C2C12 cells were cultured in proliferation media (DMEM supplemented with 4.5 g/L D-glucose, L-glutamine, penicillin-streptomycin, and 20% FCS). Cells were passaged at 60% of confluence. Spontaneous fusion of some myoblasts in sub-confluent cultures served a signal to induce differentiation. In order to stimulate myogenesis, the proliferation media was replaced with differentiation media (DMEM media supplemented with 4.5 g/L D-glucose, L-glutamine, penicillin-streptomycin, 2% of horse serum). Differentiation media was replaced every day, RNA samples were collected for experiments at day 0 and then on the second and fourth day after induction or as indicated in the figure legend.

### 2.2. Morphological Features Determination

To determine the morphological features of differentiated myotubes the myotubes were visualized with MitoTracker dye and nuclei were counterstained with DAPI: the MitoTracker dye (Mito Traker Red CMXRos, M7512, ThermoFisher Scientific, Waltham, MA, USA) was added to the living cells at concentration of 250 nM and incubated for 15 min at 37 °C. Then the dye was washed out with PBS and fixed with a 4% paraformaldehyde for 10 min at room temperature. Nuclei were visualized by DAPI staining. The representative numbers of pictures (indicated in the figure legend) were taken for each tested condition. The fusion coefficient was determined as the ratio of the nuclei number in myotubes formed by three or more nuclei to the total number of nuclei on the picture. The width of the myotubes was determined using ZEN Blue software (Carl Zeiss Microscopy, Jena, Germany).

### 2.3. Plasmids and Mutagenesis

Mammalian expression lentiviral vector with human *LMNA* cDNA insert pCDHblast MCSNard OST-*LMNA* (plasmid #22661) was obtained from Addgene (Watertown, MA, USA). Site-directed mutagenesis of whole plasmid strategy was employed: a PCR step with primers bearing the mutation, followed by a DpnI digestion step to digest the methylated parental/wild-type plasmid, and transformation into competent cells for nick repair. Primers containing the desired mutations are presented in Table 1 NM_170707 mRNA was used as a template for primers design. The process is described below in details.

The PCR reaction mixtures contained 5 µL of 5× reaction buffer, 1.25 µL of each primer (10 µM), 1 µL of 10 mM dNTP mix, 50 ng of dsDNA template, 1 µL of DMSO (Dimethyl sulfoxide), 0.5 µL of Q5 Phusion^®^ high-fidelity DNA polymerase (2000 units/mL; New England Biolabs, Ipswich, MA, USA), and nuclease-free water to a final volume of 25 µL. The PCR conditions were: denaturation at 98 °C for 30″, followed by 30 cycles of amplification: denaturation at 98 °C for 10′, annealing at 60 °C for 20″, and primer extension at 72 °C for 5′20″. Fifteen microliters (15 µL) of each reaction were taken to test the quality of the amplification product by electrophoresis (1% TAE agarose gel). The remaining products were treated with methylation-sensitive restriction enzyme DpnI at 37 °C for 30 min to remove the methylated original plasmid from the mix before transforming into *E. coli*: each reaction (total volume of 10 µL) consisted of 8 µL of PCR reaction, 1 µL of 10× FastDigest buffer (Thermo Scientific, Wilmington, DE, USA), and 1 µL of FastDigest DpnI restrictase (Thermo Scientific, Wilmington, DE, USA).

After 5 min of enzyme heat inactivation at 80 °C the mutated plasmids were transformed into XL-1 Blue *E. coli* competent cells (#CC001, Evrogen, Moscow, Russia) using the heat shock method. Specifically, 5 μL of the DpnI digestion products and 100 μL of competent cells were mixed and incubated for 30 min on ice, then heated at 42 °C for 45″, and placed back on the ice for 2 min. The transformed bacteria were incubated in LB media on the shaker for 1 h at 37 °C following by overnight plating on selecting agar media (with ampicillin, 100 μg/mL) at 37 °C. The colonies were analyzed for efficiency of mutagenesis: DNA sequencing of the newly-synthesized mutated plasmids was performed using the primers listed in the Table 1 and Table 2.

### 2.4. Lentivirus Production, Infection, and Establishment of Stable Cell Lines

Lentiviruses were generated by transient co-transfection of HEK 293T cells with the transfer vector and the helper plasmids, using linear polyethylenimine hydrochloride (PEI). Lentiviral packaging plasmids were a gift of D. Trono (École Polytechnique Fédérale de Lausanne, Switzerland) [29]. PEI was obtained from Polysciences (PEI MAX 40K, 24765-1, Warrington, PA, USA), and stock solution of 1 mg/mL was prepared according to the supplier’s instructions. HEK293T cells cultured in 100-mm culture plates up to 60–80% confluence. One hour prior to transfection the culture medium was changed.

293T cells were grown 100-mm dishes up to 60–80% and co-transfected with 15 µg of expression vector pCDHblast MCSNard OST-*LMNA*, 5.27 µg of envelope plasmid pMD2.G, and 9.73 µg of packaging plasmid pCMV-dR8.74psPAX2. PEI and pDNA were diluted to equal volumes with Opti-MEM^®^I reduced-serum medium (GibcoTM) prior to use and mixed by adding PEI into pDNA at a ratio of 1:2. The mixture incubated at room temperature for 15–20 min and added dropwise to the HEK293T cells. The next day the medium was replaced, and the cells cultured for 24 h for virus production. After that the conditioned medium was collected, centrifuged at 1000 rpm for 5 min at room temperature, filtered through 0.45 µM PVDF membrane filters, and concentrated by ultracentrifugation at 20,000× *g* for 2 h. The resulting pellet was resuspended in 1% BSA/PBS and frozen in aliquots at −80 °C.

Concentrated viral particles were used then for C2C12 myoblasts modification. For that, viral particles were added to freshly seeded C2C12 cells in DMEM containing 10% FBS and 8 μg/mL polybrene (hexadimethrine bromide; H9268, Sigma-Aldrich, St. Louis, MO, USA). Six hours the medium was replenished with DMEM + 20% FBS. After 48 h C2C12 myoblasts were passaged, and transduced cells were selected for successful infection using 10 µg/mL blasticidin (15205, Sigma-Aldrich, St. Louis, MO, USA). Culture medium with blasticidin was replaced every day with occasional trypsinization of cells when they reached 80% confluency to gradually remove dead or dying cells. Non-infected C2C12 cells were used as a control. Upon complete cell death in the controls (usually after a week of selection) antibiotic-resistant populations were expanded and used directly in experiments or placed into liquid nitrogen.

### 2.5. Library Construction and RNA-Seq

Total RNA was extracted with ExtractRNA reagent (#BC032, Evrogen, Moscow, Russia) and quantified using Qubit 2.0 fluorometer (Assay Kit Q32852, Life Technologies, Carlsbad, CA, USA). One microgram (1 μg) of RNA was used for library synthesis with an Illumina TruSeq Stranded mRNA Sample Preparation kit (20020594, Illumina, San Diego, CA, USA). Three independent biological replicates were used for RNA-seq in each experiment. Libraries were constructed according to the instructions provided by the manufacturer.

The libraries were quantified using Qubit dsDNA HS Assay Kit (Q32854, Life Technologies, Carlsbad, CA, USA). The quality of the library was assessed with Agilent High Sensitivity DNA kit on a Bioanalyzer 2100 system (Agilent Technologies, Palo Alto, CA, USA). The libraries were multiplexed, clustered, and sequenced on an Illumina HiSeq 2500 (TruSeq v3 chemistry) as described by the manufacturer using the 1 × 50-bp single-end read mode with an eight-base index barcode read.

### 2.6. Relative Quantification of mtDNA Copy Number Using Real-Time PCR

To determine the relative mtDNA copy number a real-time polymerase chain reaction was employed and corrected by simultaneous measurement of the nuclear DNA as described [30]. Total DNA from C2C12 cells was isolated by FlexiGene DNA kit (51206, Qiagen, Venlo, The Netherlands) according to the manufacturer’s instructions. NanoDrop 1000 Spectrophotometer was used to test the concentration and quality of DNA (Thermo Scientific, Wilmington, DE, USA). RT-qPCR was performed on an Applied Biosystems 7500 (Applied Biosystems, Foster City, CA, USA). Kcnj13 (inward rectifier potassium channel 13) gene was used as the nuclear reference gene (nDNA). Primers sequences for murine mitochondrial fragment and Kcnj13 are given in a Table S1 [31]. SYBR + Low ROX PCR Master Mix (PK156L, Evrogen, Moscow, Russia) was used for RT-qPCR. All reactions were checked on the agarose gel after performing quantitative PCR. The real time PCR results were calculated in the term of threshold cycle (Ct) values. mtDNA copy number was determined for each sample relative to the single copy of nuclear gene by comparative Ct method using the equation:

ΔCt = Ct nDNA − Ct mtDNA

Relative mitochondrial DNA content = 2 × 2 ΔCt

### 2.7. Cell Respiration

Cell respiration was measured using an XFe24 Analyzer (Agilent Technologies, Santa Clara, CA, USA) that allows the determination of oxygen consumption rates (OCR) and extracellular acidification rates (ECAR) in real-time. Twenty thousand cells per well were seeded in a 24-well microplate (Agilent Technologies, Santa Clara, CA, USA), cultured until sub-confluence, then induced to differentiate. Before the assay, a microplate was incubated for 60 min without CO_2_ in Seahorse XF Base Medium. The OCR/ECAR measurements performed under basal conditions and after the addition of ATP synthase inhibitor oligomycin (2 μM); a mitochondrial protonophore uncoupler (N5,N6-bis(2-fluorophenyl)-[1,2,5]oxadiazolo[3,4-b]pyrazine-5,6-diamine) BAM15 (10 μM); inhibitors of electron transport chain complexes I and III rotenone (1 μM), and antimycin A (1 μM) respectively. Data were normalized to the protein content.

Following parameters were calculated: non-mitochondrial oxygen consumption = (minimal OCR after rotenone/antimycin A injection); basal respiration = (last OCR before oligomycin injection) − (non-mitochondiral OCR); proton leak = (basal respiration) − (OCR due to ATP production) − (non-mitochondiral OCR); maximal respiration = (maximal OCR after BAM15 injection) − (non-mitochondiral OCR); ATP production = (last OCR before oligomycin injection) − (minimal OCR after oligomycin injection); coupling efficiency = (ATP production)/(basal respiration) × 100; spare respiratory capacity as a % = (maximal respiration)/(basal respiration) × 100; cell respiratory control ratio = (maximal respiration)/(proton leak); OCR/ECAR ratio = the highest of the three OCR values divided by the corresponding ECAR value. Statistical analysis was performed using GraphPad Prism version 8.4.1 for Windows (GraphPad Software, San Diego, CA, USA, www.graphpad.com).

### 2.8. Glycolysis Stress Assay

Glycolytic function of C2C12 myotubes was measured using a Seahorse XF Glycolysis Stress Test Kit with a Seahorse XFe24 Analyzer (Agilent Technologies, Santa Clara, CA, USA) according to manufacturer’s instructions. Nondifferentiated C2C12 myoblasts were seeded at a density of 20,000 cells/well onto a Seahorse XF24 microplate (Agilent Technologies, Santa Clara, CA, USA), and differentiated myotubes were prepared. The culture medium was replaced for the assay medium (Seahorse XF Base Medium, pH 7.4, supplemented with 1 mM L-glutamine), and the microplate was placed in a non-CO_2_ incubator at 37 °C for one hour before the assay. Glycolytic activities were assessed by measuring of the extracellular acidification rates (ECAR). ECAR was measured under basal conditions, when the myotubes were incubated in glucose-free media followed by the glycolysis induction (10 mM glucose), 2 µM oligomycin treatment (induction of maximal glycolysis due to ATP synthase inhibition) and, finally, 50 mM 2-deoxy-D-glucose (glycolysis inhibition).

Basal ECAR = non-glycolytic acidification. After addition of glucose, glycolysis was calculated by subtracting of non-glycolytic acidification ECAR from the highest ECAR value. Following injection of oligomycin, glycolytic capacity was calculated as the basal ECAR subtracted from the highest ECAR used to meet cellular demands after ATP synthase is inhibited. The difference between glycolytic capacity and glycolysis rate defines glycolytic reserve. The mean values from 6–8 replicate wells were recorded and presented as the mean ± standard error of the mean (SEM). After the assay, the supernatant was removed, and RIPA lysis buffer containing protease inhibitor cocktail (cOmpleteTM, 11873580001, Roche, Basel, Switzerland) was added to the cells. The protein concentration was determined using DCTM Protein Assay Kit (500-0112, Bio-Rad, Hercules, CA, USA). The ECAR values were normalized to the amount of total protein.

### 2.9. Cell Proliferation Assay

C2C12 mouse myoblasts transduced with WT-h*LMNA*, G232E-h*LMNA*, and R482L-h*LMNA* were seeded into six-well culture plates at a density of 20,000 cells per well, and cultured at 37 °C in a 5% CO_2_ incubator. The cells were harvested with trypsin/EDTA solution and counted after digestion every 24 h for three days. Four separate wells were counted per time point for each cell line. To calculate the cell population doubling time (PDT) the equation was used:

PDT = t × [lg2/(lgNt − lgN0)]

where t is the time for cell culture (unit: h), N0 is the initial cell number, and Nt is the harvested cell number. Results were averaged, and the standard deviation was calculated. Statistical significance was determined by Student’s unpaired *t*-test. *p* < 0.05 was considered significant.

### 2.10. Western Blotting

Cells were lysed with buffer containing 50 mM Tris (pH 8.0), 150 mM NaCl, 1% Triton X-100, 0.5% sodium deoxycholate, 0.1% SDS, 5 mM EDTA, and protease inhibitor cocktail (cOmpleteTM, 11873580001, Roche, Basel, Switzerland). Extracts were separated by sodium dodecyl sulfate-polyacrylamide gel electrophoresis (SDS-PAGE) and then transferred to 0.45 µm pore size nitrocellulose membrane (1620115, Bio-Rad, Hercules, CA, USA). Western blot was performed using standard procedures, with blocking in 5% skimmed milk for 1 h and washes in PBS containing 0.05% Tween 20. Membranes were probed at 4 °C overnight with target antibodies: Anti-skeletal myosin (FAST) (M4276, Sigma-Aldrich, St. Louis, MO, USA), anti-myosin (Skeletal, Slow) (M8421, Sigma-Aldrich, St. Louis, MO, USA), and myosin heavy chain (MF20, R&D Systems, Minneapolis, MN, USA). The blots were revealed by secondary HPR-conjugated antibodies (#1706516, Bio-Rad, Hercules, CA, USA), and chemiluminescense was detected using Fusion Fix imaging system (Vilber Lourmat, Marne La Vallee, France), and analyzed with FusionCapt Advance FX7.

### 2.11. Immunocytochemistry

Cells were grown on cover glasses or in culture dishes (if differentiation was planned), fixed in 4% paraformaldehyde for 10 min at 4 °C (myoblasts) or with methanol for 20 min at −20 ° C (myotubes) and then permeabilized with 0.02% Triton X-100 for 5 min. To block the nonspecific binding cells/myotubes were incubated for 30 min with 15% FCS. One hour incubation with primary antibodies: myosin heavy chain (MF20, MAB4470, R&D, USA); lamin A (Leica, Wetzlar, Germany) (*LMNA*, Leica, Wetzlar, Germany) was performed. The secondary antibodies conjugated with Alexa Fluor 546/Alexa-488 (Molecular Probes, Eugene, OR, USA) were added for 45 min at room temperature in the dark. Nuclei were counterstained with DAPI (Molecular Probes, USA).

### 2.12. MitoTracker Staining

The Mito Traker dye (Mito Traker Red CMXRos, M7512, ThermoFisher Scientific) was added to the living cells at concentration of 250 nM and incubated for 15 min at 37 °C. Then the dye was washed out with PBS and fixed with a 4% PFA for 10 min at RT followed by DAPI staining.

### 2.13. Q-PCR

Total RNA was purified with ExtractRNA reagent (Evrogen, cat.no. BC032, Russia). cDNA was made from 500 ng of total RNA using a MMLV RT kit (Evrogen, SK021, Russia). A quantitative evaluation of gene expression was performed with qPCR mix-HS SYBR + ROX (Evrogen, cat. no. PK156, Russia). mRNA normalized to Gapdh expression. Sequences for Q-PCR primers available in Supporting Materials Table S4.

### 2.14. Processing of RNA-Seq Data

Raw data were gotten in FASTQ format from Illumina HiSeq 2500 (Illumina, san Diego, CA 92122, USA), the quality of RNA-seq data was evaluated using FastQC tool (v0.11.5) (FastQC: a quality control tool for high throughput sequence data. Andrews S. (2010). Available online at: http://www.bioinformatics.babraham.ac.uk/projects/fastqc), and the read length was 50 bp. Filtering and adapter trimming were made using fastp tool (v0.20.0) [32]. Reads were mapped to the mouse genome using aligner STAR v2.604a [33] with reference genome GRCm38.p6 primary assembly and annotation GENECODE release M22. Mapped reads were count with the featureCounts program v.1.6.4 [34]. The top 14,321 most expressed genes were chosen after filtering, quantile, and logarithmic normalization of counts for differential expression analysis. Differentially-expressed genes (DEGs) were determined using R package DESeq2 [35] with pairwise comparison of conditions. Genes *p*-values were adjusted using the Benjamini–Hochberg procedure and filtered with false discovery rate control (FDR) = 0.01, only genes with log2 fold change > 1 were considered as differentially expressed. To find significantly enriched pathways gene set enrichment analysis (GSEA) was made using fgsea R package [36]. Additionally, hypergeometric test was performed to recognize the significance of crossing DEGs with gene sets using MSigDB [37] online tool. Pathway gene sets were taken from the Gene Ontology biological processes collection, hallmark MSigDB, and the KEGG PATHWAY metabolism database; significantly enriched pathways were filtered with an FDR level of 0.05. Raw sequence data and the read count table are available at the Gene Expression Omnibus repository under accession number GSE150365.

## 3. Results

### 3.1. *LMNA* Mutations Affect Both Proliferation Activity and Myogenic Commitment in Transgenic C2C12 Myoblasts

**Figure 1:**
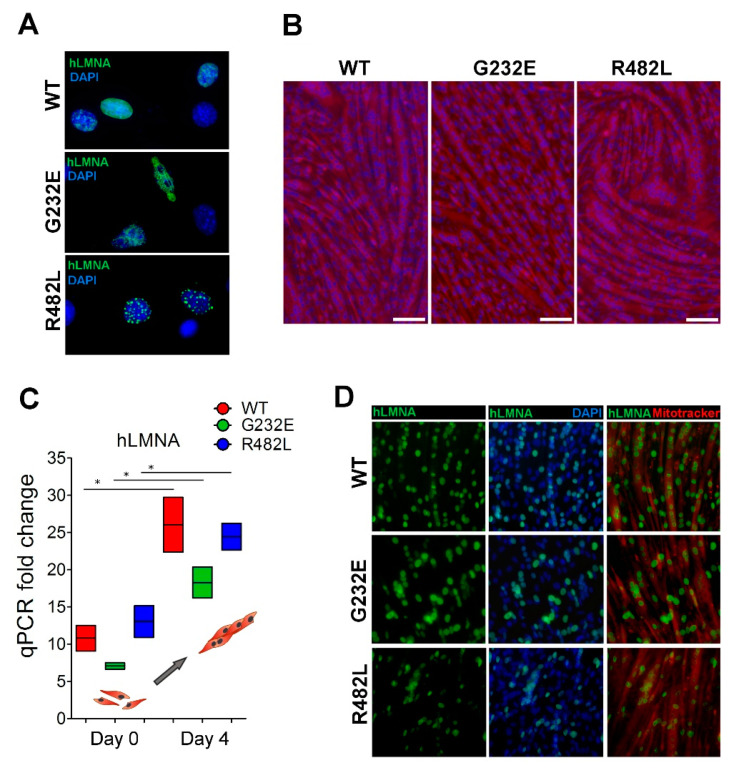
Validation of experimental model: (**A**) Verification of transgene h*LMNA* expression in C2C12 mouse myoblasts: the cells were stained with anti lamin A antibody recognizing only human lamin A. *LMNA* G232E/R482L shows aggregates and nuclear blebbing in some of the transduced cells as a result of the mutation; (**B**) stimulation of myogenic differentiation resulted in myotubes formation in all transgene lines (scale bar = 200 μm); (**C**) h*LMNA* mRNA expression tested in transgene C2C12 myoblasts and in differentiated myotubes (*n* = 3; * *p* < 0.05); (**D**) nuclei in myotubes demonstrate h*LMNA*-positive staining (scale bar = 100 μm).

Mouse myoblast cell line C2C12 transducted with lentiviruses, bearing either wild-type or mutant G232E/R482L-h*LMNA*s, was used for all experiments. Staining of transducted cells with antibody specifically recognizing human *LMNA*, but not mouse *LMNA*, allowed us to control the efficiency of transduction in all experimental samples used for the analysis. We have found that mutant G232E/R482L-*LMNA* formed aggregates and promoted nuclear blebbing (Figure 1A) similar to previously published reports [25,38,39,40,41]. We further proved the ability of all transducted myoblasts successfully differentiate into myotubes (Figure 1B) and demonstrated that h*LMNA* mRNA expression levels increased significantly in differentiated myotubes compared to non-differentiated myoblasts as expected (Figure 1C) [42]. Additionally, we proved that all h*LMNA*-positive myoblasts successfully underwent fusion and formed myofibers in all transgenic lines (Figure 1D). The percentage of *LMNA*-positive cells in differentiated myotubes was similar in all lines (Figure S5).

**Figure 2:**
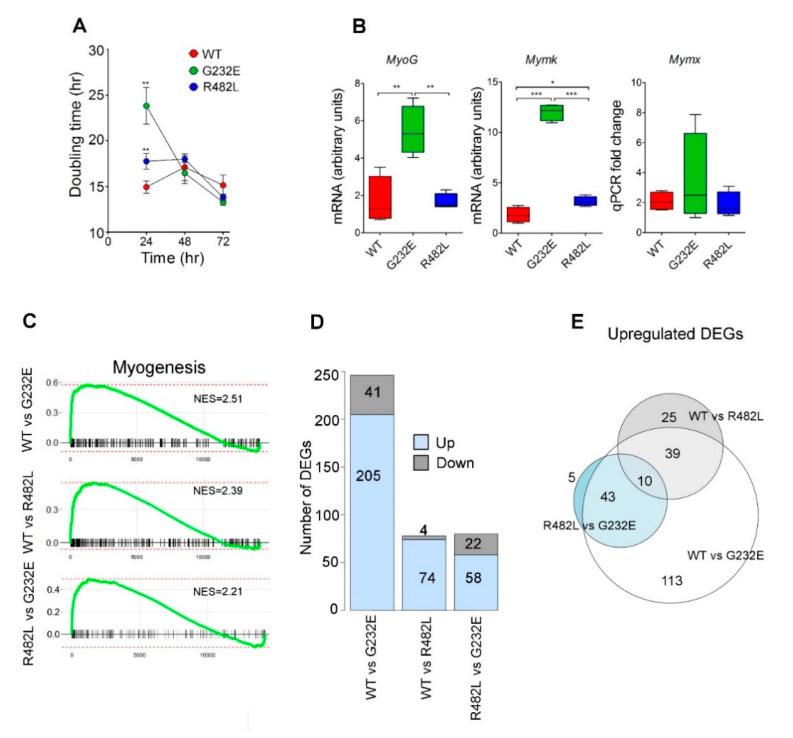
G232E/R482L-*LMNA*—induced alterations in functional properties of C2C12 myoblasts: (**A**) Estimation of the cell population doubling time for C2C12 cell lines, transduced with WT-*LMNA*, G232E-*LMNA*, and R482L-*LMNA* at three time points until 72 h. Values are mean ± SEM (*n* = 4). ** *p* < 0.01; (**B**) mRNA expression of the genes that regulate the early steps of myogenic differentiation in C2C12 myoblasts. (*n* = 4; * *p* < 0.05; ** *p* < 0.01; *** *p* < 0.001); (**C**) GSEA enrichment plots of the hallmark myogenesis pathway over ranked genes between all possible pairs of C2C12 myoblasts: WT versus G232E-*LMNA*, WT versus R482L-*LMNA*, and R482L-*LMNA* versus G232E-*LMNA*. (*p*-adjusted < 0.001, normalized enrichment scores (NES) are shown); (**D**) number of up- and downregulated differentially expressed genes (DEGs) found in all three pairs (log2 fold change > 1, *p*-adj < 0.01); (**E**) Venn diagrams of upregulated DEGs.

The differentially-expressed genes (DEG) were analyzed between all possible pairs of cell lines, (Figure 2D,E) following by identification of biological functions associated with DEGs. We found that the substantial proportion of upregulated genes both in WT/G232E and WT/R482L pairs (Figure 2D) belong to the pathways that regulate myogenesis (as shown in a Table 3). Further, when analyzing the DEGs in R482L/G232E pair we revealed that G232E-*LMNA* cells were also characterized by upregulation of myogenesis signaling pathways (Table 3). The details of the analysis and the full list of pathways which were significantly different between transgene lines, and genes which expression was altered in G232E-*LMNA* and R482L-*LMNA* mutants is given in the extended data presented in Supplementary Figure S1 (Principal component analysis (PCA) plots of 24 samples) and Figure S2 (Heat map of normalized read counts for 24 samples and 14,321 genes) and Supplementary Table S2 (Full list of differential-expressed genes) and Table S3 (Full list of pathways that were significantly different between transgene lines).

The analysis of proliferation activity of transgenic C2C12 myoblasts showed the significant difference between WT and mutant lines. At 24 h after seeding both mutant cell lines demonstrated diminished proliferation rate compared to WT cells with G232E-*LMNA* cells showing the lowest proliferation capacity and the highest doubling time (Figure 2A).

We hypothesized that the substantial delay in proliferative activity of G232E/R483L-*LMNA* myoblasts might be linked to the quick transition to the differentiation commitment caused by mutant lamins. In order to test this hypothesis, we examined the expression of genes that regulate early steps of myogenesis: myogenic transcription factor *MyoG*, myoblasts fusion factors Myomaker (*Mymk*), and Myomixer (*Mymx*). We detected the upregulated expression of *Mymk*, but not *Mymx* and Myog, both in G232E and R482L-*LMNA* cells (Figure 2B). To further confirm this hypothesis, we used RNA sequencing approach and revealed a significant upregulation of myogenesis pathway in both G232E-*LMNA* and R482L-*LMNA* samples compared to WT-*LMNA* in the absence of differentiation stimuli. Of note, G232E-*LMNA* samples demonstrated significantly higher myogenesis upregulation compared to R482L-*LMNA* (Figure 2C).

To summarize, we have shown that C2C12 myoblasts harboring G232E-*LMNA* and R482L-*LMNA* exhibit decreased proliferation capacity and increased expression of genes associated with muscle differentiation program even in the absence of differentiation stimuli.

### 3.2. *LMNA* Mutations Affect Differentiation Efficacy and Dynamics in Transgenic C2C12 Myoblasts

**Figure 3:**
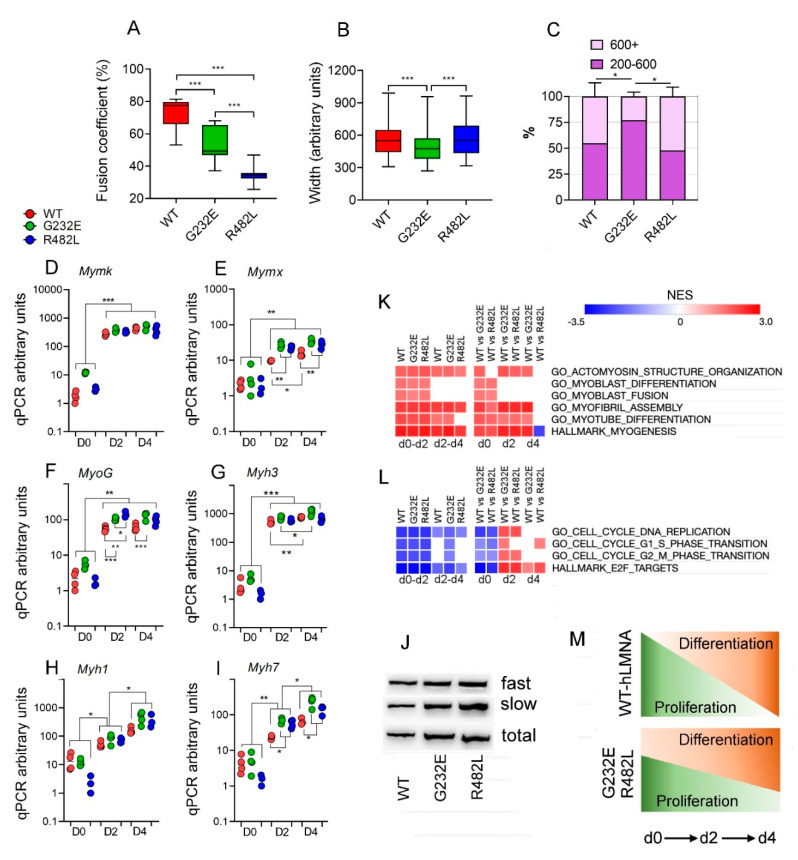
Myogenesis in transgene cultures. (**A**) Fusion coefficient calculated as a percent of nuclei incorporated in differentiated myotube in independent photographs; *n* = 8; *** *p* < 0.001, *t*-test; (**B**) width of myotubes, measured in arbitrary units in 5–6 independent photographs; the number of myotubes on each photo was 10–20; *** *p* < 0.001, *t*-test; floating bars are shown with mean, minimum and maximum values; (**C**) diagram illustrate the fractions of “massive” and “overage” myotubes in cultures; * *p* < 0.05; (**D**–**F**) The mRNA expression dynamics of genes that regulate myoblast fusion (*Mymk*, *Mymx*) and of myogenic regulatory factor *Myog* (*n* = 4; mean + SEM; * *p* < 0.05; ** *p* < 0.01; *** *p* < 0.001; Mann–Whitney test); (**G**–**I**) the mRNA expression dynamics of fiber-specific genes: fast glycolitic fiber Myh1 isoform; oxidative slow fiber Myh7 isoform; embryonic myosin isoform Myh3; (*n* = 4, mean + SEM, * *p* < 0.05; ** *p* < 0.01; Mann–Whitney test). (**J**) Western blotting analysis for total myosin (slow (antibody interact with slow *Myh7*) and fast (antibody interact with fast *Myh1/Myh2*) proteins in cultures stimulated with myogenic differentiation media at day 4 after induction; (**K**,**L**) Results of gene set enrichment analysis visualized as a heat map of normalized enrichment score (NES) that display significant up/downregulated myogenic pathways (**K**) and cell cycle dynamics (**L**); (**M**) summarized diagram that illustrates the considerable dysregulation in coordination of cell cycle dynamics and myogenic differentiation in G232E/R482L-*LMNA* myoblasts.

All lines of transgenic myoblasts responded very well to the differentiation stimuli (Figure 1B). Yet, we have detected some important morphological differences: fusion coefficient calculated as a present of nuclei incorporated into myotubes was the highest in WT-*LMNA* line and the lowest in R482L-*LMNA* line (Figure 3A). Although the range in width was similar in all samples (Figure 3B), the overage width of myotubes was significantly smaller in G232E-*LMNA* line (Figure 3B) as well as the fraction of large massive myotubes (Figure 3C).

The mRNA expression of muscle-specific genes, *Mymk* and *Mymx*, which regulate myoblasts fusion during early steps of myogenesis [43,44] did not correlate with decreased fusion coefficient in R482L/G232E lines: *Mymk* expression substantially increased in all samples during transition from day 0 to day 2 and stabilized at the same level up to the end of experiment (Figure 3D); the expression of the *Mymx* reached the maximum by the day 2 in both mutant lines and showed the small but significant increase from day 2 to day 4 in WT line. Surprisingly, the levels of *Mymx* expression were substantially higher in mutant lines than in WT line (Figure 3E). The expression patterns of myogenic regulatory factor *Myog* that controls the transition from early to late differentiation steps [45,46] was similar to one of *Mymx* with stabilization by day 2 and increased levels in mutant lines (Figure 3F). The mRNA expression of different myosin isoforms showed two different patterns (Figure 3G–J): the expression of embryonic *Myh3* isoform increased by day 2 in all samples, and then stabilized at similar levels (Figure 3G), whereas the mRNA expression of adult fast glycolytic *Myh1* and slow oxidative *Myh7* myosins showed well-defined two-step increase of expression levels with the expression of slow isoform significantly higher in mutant lines (Figure 3H,I); Western blot analysis confirmed the mRNA expression patterns of *Myh1/Myh7* at the protein level (Figure 3J).

To elucidate the mutation-specific response to stimulation of myogenesis we performed an analysis of transcriptomes in the time course of differentiation. For each transgenic cell line we determined genes differentially-expressed during early (day 0 to day 2) and late (day 2 to day 4) steps of myogenic differentiation. Then we have determined the signaling pathways that differentially-expressed genes belong to, and performed gene set enrichment analysis (GSEA) to find significantly enriched pathways. Both differential expression analysis and GSEA showed the same enrichment (Figure 3K,L).

As seen from Figure 3K (left), in all transgenic samples during transition from day 0 to day 2 we detected the upregulation of pathways that control different aspects of skeletal muscle development including myoblasts differentiation and fusion, myofibril assembly, muscle contraction apparatus development (actomyosin structure organization), myotube differentiation and development. During the late phase of differentiation, the pathways that regulate the myoblasts differentiation and fusion were stabilized, confirming the above described data for the expression of specific genes (Figure 3D–F,I). During the transition from day 2 to day 4 in all samples we detected the upregulation of pathways that control skeletal muscle fiber maturation (Figure 3K, left). Thus, at the level of the transcriptome analysis, we have confirmed that all transgene lines show similar abilities to control myogenesis with well-distinguished early and late phases of muscle differentiation.

When comparing the mutant and WT cell lines in their ability to transit through successive stages of myogenesis the significant alterations in the mutant cell lines were detected (Figure 3K, right). First, in good agreement with the data above (Figure 2C, Table 1), we have detected the upregulation of myogenic pathways in myoblasts bearing both mutant *LMNA*s (Figure 3K, right, day 0). Second, we have found that while pathways that control early steps of myogenesis, like myoblast fusion and differentiation, did not differ between all transgenic lines, the pathways that control fiber formation and maturation were upregulated in both mutant lines even at the early steps of myogenesis. These pathways remained upregulated in G232E-*LMNA* but not in the R482L-*LMNA* up to the end of the experiment.

Cell differentiation and proliferation usually show inverse relationship and terminal differentiation coincides with permanent exit from the division cycle. Therefore, we hypothesized that the described differences between WT and G232E/R482L-*LMNA* mutant lines might be related to the dyscoordination between cell cycle exit and differentiation. To test this hypothesis, we checked out the dynamics of signaling pathways that control cell cycle in transgene lines during differentiation. Indeed, we detected the inversed relationship between pathways that stimulate myogenesis and cell cycle progression: pathways that control DNA replication and E2F target genes both required for cell cycle activity [47] were continuously downregulated in all lines from day 0 up to day 4; however, pathways that control G1/S and G2/M transition were stabilized by day 2 in WT and R482L-*LMNA* lines but not in G232E-*LMNA* (Figure 3L, left). Furthermore, when we compared the activity of cell cycle pathways between lines at each time point, we detected downregulation of pathways that activate cell cycle progression in mutant proliferating myoblasts and substantial upregulation of these pathways in mutant cells after stimulation of myogenesis at both early and late stages of differentiation (Figure 3L, right). Together, these data indicate the considerable dysregulation in coordination of cell cycle dynamics and myogenic differentiation in mutant transgenic C2C12 myoblasts harboring G232E-and R482L-*LMNA* mutations (Figure 3M).

### 3.3. *LMNA* Mutations Affect Cellular Bioenergetics of C2C12 Myotubes

Myogenic differentiation is highly coordinated with regulation of energy metabolism, therefore, we hypothesized that dyscoordination in the differentiation program in C2C12 myoblasts bearing G232E/R482L-*LMNA* mutations can be accompanied by alterations in the metabolic status of G232E/R482L-*LMNA* myotubes.

To investigate the general effect of mutant *LMNA* expression on cellular bioenergetics, we employed Seahorse technology: mitochondria and glycolysis stress tests were performed on C2C12-differentiated myotubes expressing WT-*LMNA*, G232E-*LMNA*, and R482L-*LMNA*. This technique allows to quantify OXPHOS and glycolysis by using specific protocols designed to dissect distinct components with pharmacological agents.

**Figure 4:**
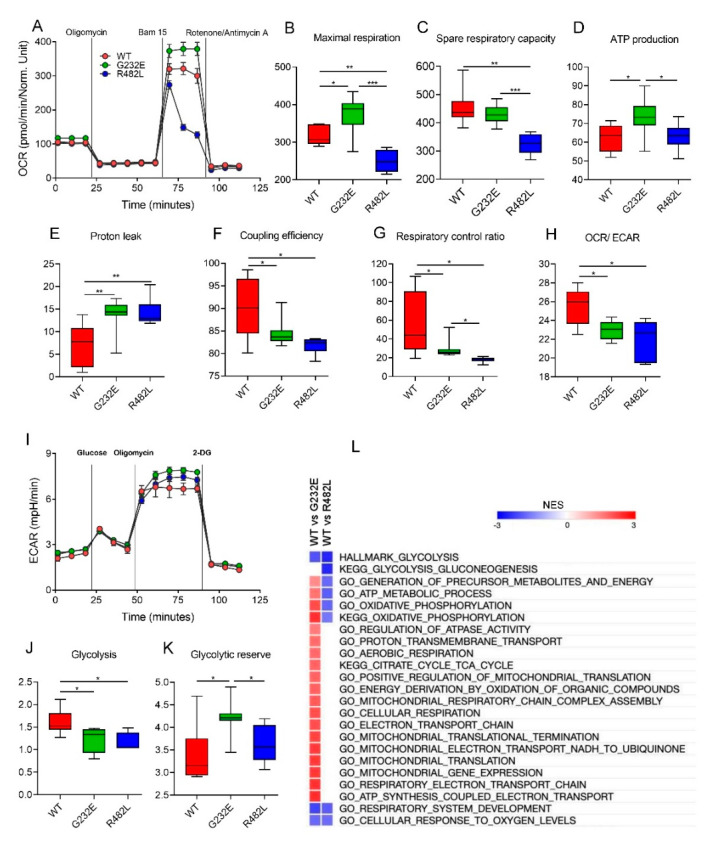
Metabolic profiles of C2C12 myotubes bearing lentiviral WT/G232E/R482L-*LMNA*. (**A**–**H**): Determination of mitochondrial bioenergetic parameters from OCR profile. (**A**) OCR traces for myotubes expressing WT/G232E/R482L-*LMNA*; (**B**–**G**) Indices of mitochondrial respiratory function: (**B**) maximal respiratory capacity; (**C**) spare respiratory capacity; (**D**) ATP-linked respiration; (**E**) proton leak respiration; (**F**) coupling efficiency; (**G**) cell respiratory control ratio; (**H**) OCR/ECAR ratio at maximal respiration. OCR was measured at the same time as ECAR. (**I**–**L**): Determination of glycolytic function. (**I**) Kinetic profiles of ECAR in myotubes expressing WT/G232E-*LMNA*/R482L-*LMNA*. ECAR was measured in real time, as indicated. Indices of glycolytic pathway activation calculated from ECAR profile: (**J**) Maximal glycolysis and (**K**) glycolytic reserve capacity. All data are calculated from 6–8 Seahorse microplate wells and are normalized to total protein in each well. * *p* < 0.05; ** *p* < 0.01 *** *p* < 0.001; two-tailed Student’s *t*-test. All floating bars are shown with mean, minimum and maximum values. (**L**) Heat map of NES values after GSEA analysis represents metabolic pathways found up- or down-regulated in G232E/R482L myotubes compare with WT-*LMNA*; FDR = 0.05.

Real-time measurements of oxygen consumption rate (OCR), an indicator of mitochondrial respiration, are shown in Figure 4A. Maximal respiration and spare reserve capacity (Figure 4B,C) were found to be the lowest in R482L-*LMNA* line. These myotubes were unable to maintain maximal OCR levels following the addition of the uncoupler BAM15, with the OCR decreasing to basal levels even prior to rotenon/antimycin A treatment (Figure 4A) which implies that at basal levels, these myotubes were operating closer to maximal OCR capacity, and any increase was unsustainable, resulting in a lower reserve capacity.

On the contrary, in G232E-*LMNA* line the maximal rate of respiration and ATP-linked respiration were significantly higher than in two others (Figure 4C,D), which, along with induced basal OCR (Figure 4A), suggests enhanced ATP turnover and demand. Strikingly, a G232E-related increase in basal and maximal OCR was accompanied by a significantly induced proton leak (Figure 4E). Proton leak is the predominant mechanism responsible for the incomplete coupling of substrate oxidation and ATP synthesis, and its increase was observed in both mutant transgene lines (Figure 4E). Subsequent analysis of the respiratory flux control ratios provided further confirmation of potential mitochondrial dysfunction in the mutant *LMNA* lines. Coupling efficiency (fraction of basal mitochondrial oxygen consumption used for ATP synthesis) and respiratory control ratio (index that represents the mitochondrial coupling state) were found to be significantly lower in both mutant lines compared to WT-*LMNA* (Figure 4F,G). Together, these data might indicate the uncoupling effect of *LMNA* mutations on mitochondrial respiration and decrease in the efficiency of oxidative phosphorylation.

Given that the myotube is a highly metabolically active cell type that relies heavily on OXPHOS we next determined the ratio OCR/ECAR (extracellular acidification) to assess the relative contribution of glycolysis and mitochondrial respiration to energy generation [48]. A higher OCR/ECAR ratio indicates that energy is mainly generated through mitochondrial OXPHOS, whereas a lower OCR/ECAR ratio indicates that energy is predominantly generated by glycolysis. The OCR/ECAR ratio showed preference for OXPHOS in the WT-*LMNA* compared with G232E/R482L-*LMNA* myotubes (Figure 4H), indicating that the mitochondria of the WT-*LMNA* myotubes have a greater potential for substrate oxidation and ATP turnover. To make sure that observed distinctions in respiration were not the consequence of altered mitochondrial content in the myotubes with the mutant transgenes we determined the ratio of mtDNA to nuclear DNA and did not detect any differences between the cell lines neither in the myoblasts nor in the myotubes (Figure S3).

To complete the bioenergetics analysis, we performed a glycolysis stress test. The real-time measurements of ECAR are shown in Figure 4I. Myotubes bearing G232E/R482L-*LMNA* exhibited lower glycolytic rates compared to WT-*LMNA* line (Figure 4J), while G232E-*LMNA* myotubes were found to have the highest glycolytic reserve, the cellular ability to increase the glycolytic rates upon growing energy demand (Figure 4K). Importantly, glycolytic capacity (max ECAR—basal ECAR) did not differ between lines (Figure S4). Together, these findings point to the altered metabolic properties of G232E-*LMNA* and R482L-*LMNA* lines when comparing with WT-*LMNA*.

The comparative analysis of signaling pathways that regulate cellular bioenergetics in differentiated myotubes also revealed multiple significant alterations in mutant transgene lines, and these findings provide good support to the results obtained in functional experiments (Figure 4L). The hallmark glycolysis pathway and pathways that regulate cellular response to oxygen levels were downregulated in both mutant lines comparing to WT line. The pathways that control different steps of aerobic respiration and oxidative phosphorylation were either upregulated in G232E line and downregulated in R482L line comparing to WT line, or upregulated in G232E line over both WT and R482L lines. To summarize, using bioenergetic profiling and transcriptome analysis we have shown here the *LMNA* mutation-specific alterations in skeletal muscle cell bioenergetics.

## 4. Discussion

The evidences that *LMNA* is expressed by skeletal muscle stem cells led to the suggestion that stem cell dysfunction might contribute to *LMNA*-associated muscular dystrophy progression through the compromised regeneration response, including altered cell cycle dynamics and chronic rounds of repair/regeneration (reviewed in [11]). Yet, the experimental verifications of this hypothesis are still limited and most studies on *LMNA*-associated muscle dystrophies focused on the role of *LMNA* in the muscle differentiation process but not on alterations in progenitor cell’s health. A recent report using a murine model of EDMD illustrated that the absence of Lamin A/C in muscle stem cells fail to maintain the stem cells self-renewal, which results in premature senescence and exhaustion of the quiescent satellite cell pool [49]. Similar observations were earlier reported for stromal progenitor cells harboring *LMNA* mutations [50]. It is proposed that *LMNA* mutations may have a cumulative influence on muscle development due to mutation-induced alterations in the formation of lamina-associated domains: slowing the exit from the cell cycle and poorly coordinated induction of terminal differentiation [15]. Our data fit well with those observations and provide further evidence of *LMNA* mutation-induced alterations in the skeletal muscle progenitor cells’ pool self-renewal. We have shown here that both G232E-*LMNA* and R482L-*LMNA* mutations induce global disruptions in the coordination of myogenesis: these mutations not only induce the decrease to proliferate and stimulate spontaneous activation of a pro-myogenic program in proliferating C2C12 mouse myoblasts, but also cause the delay in cell cycle suppression after myogenesis stimulation, which results in disturbed coordination of late steps of myocyte differentiation. We should mention here that the significant difference in the proliferation rate was confirmed at 24 h after seeding, but not after 48 h or later. We suggest that these fluctuations in the proliferation rate reflected the heterogeneity of the cellular population and dynamic changes in the samples during culturing: the fraction of cells that were spontaneously committed to differentiate proceeded through the differentiation program and did not contribute to the proliferation rate, while the fraction of cells that continued to proliferate increased with each cycle of division leading to the shift of the balance between spontaneously committed and proliferating fractions in favor of the last one. It still remains unclear how cells choose between spontaneous myogenic commitment and proliferation mode, and the further investigations are required to clarify this issue.

All alterations mentioned above were detected in both G232E-*LMNA* and R482L-*LMNA* mutant lines and our data are in many ways consistent with the specific muscular phenotypes associated with mutations. For example, in both mutant lines we detected the delay in cell cycle suppression which, in turn, could result in the chronic rounds of myoblasts proliferation without incorporation in growing myofiber [11], resulting in regeneration defects and muscular atrophy. Interestingly, though the decrease in the fusion coefficient in the R482L was substantially greater than in G232E, the comparison of morphological features showed the decrease in myotube width and the smaller fraction of large myotubes in G232E cultures, but not in R482L which can be connoted with the muscle hypertrophic phenotype reported for some FPLD patients with arg482 *LMNA* mutation [24,51].

Bioenergetic impairments induced by *LMNA* mutations in C2C12 mouse myoblasts is another important finding in this study. Muscle differentiation follows a highly ordered, temporally distinct sequence of events, and a key event is known to be the metabolic switch from glycolysis to oxidative phosphorylation, which is necessary to provide the large pool of ATP for energetic needs of contracting muscle [52]. Thus, in the course of differentiation, myotubes rely on mitochondrial respiration as their major source of metabolic energy [53]. By using the OCR/ECAR ratio as a proxy of metabolic balance [54] we revealed that G232E/R482L-*LMNA* myotubes have a lower reliance on oxidative phosphorylation pathways compared to WT-*LMNA*. We suggest that incomplete metabolic switching and altered mitochondrial bioenergy production profiles contribute to the observed dysregulation in myogenic differentiation in G232E/R482L-*LMNA* C2C12 models.

G232E and R482L-*LMNA* mutations reprogram skeletal muscle mitochondrial bioenergetics machinery in different directions. R482L-*LMNA* myotubes demonstrated the extremely low capacity for overall metabolic activity. A decrease in maximum respiratory capacity, spare reserve capacity, and coupling efficiency is a strong indicator of potential mitochondrial dysfunction. Since skeletal muscle is highly dependent on oxidative phosphorylation for energy production, decreased mitochondrial efficiency and reserve respiratory capacity sensitize the muscle to the risk of cell death under conditions when a tissue can require additional cellular energy in response to stress or increased workload. Exhaustion of the mitochondrial reserve respiratory capacity was shown to be associated with various pathological conditions, including maladaptive cardiac hypertrophy, neurodegenerative disorders, and smooth muscle cell death [55]. Loss of both maximal and reserve respiratory capacity detected in R482L-*LMNA* should be critical for skeletal muscle tissue, which is the largest organ and a major contributor to the basal metabolic rate in the body. Importantly, pathway analysis of gene expression in R482L myotubes confirmed the functional data and revealed a general reduction in expression of OxPhos genes regulating the metabolic activity of cells. This observation is consistent with microarray analysis [21] that showed down-regulation of genes involved in complex I of the respiratory chain in cultured myotubes derived from FPLD patients with *LMNA* R482W and R482Q mutations.

By contrast, the transcriptome profiles of G232E-*LMNA* myotubes showed upregulation of energy-producing pathways, including OxPhos and the TCA cycle, which is in a full accordance with the altered bioenergetic status. However, in spite of the increased bioenergy production profile, mitochondria of G232E-*LMNA* myotubes demonstrated less coupling, reflecting non-optimal efficacy of mitochondrial metabolism.

Furthermore, in our experiments we have shown the reduced power of glycolytic machinery in mutant lines using both functional experiments and transcriptome analysis. Our data correspond well to several previous reports demonstrating alterations in balance between lipid oxidation and oxidative glucose metabolism in cultured myotubes from FPLD patients with *LMNA* R482W/R482Q mutations and glycolysis dysregulations in HeLa cells with lamin A/C dysfunction as well as in fibroblasts derived from patients with laminopathies [21,56,57]. In these reports authors showed significant changes in the expression of proteins involved in cellular energy production, including a decrease of glycolytic and other metabolic enzymes and assumed that an intrinsic defect in skeletal muscle glucose metabolism is associated with mutations in lamin A.

## 5. Study Limitations

Though the lentiviral vectors are widely used in genome editing applications, the lentiviral-based experimental models may have some limitations due to the possible effects of transduction itself on the host cell properties. The experimental model used in this study was designed with the assumption that the transduction itself affects all cultures equally/in similar ways and, therefore, is suitable to study the mutation-specific effects on C2C12 myoblasts. We believe that the results obtained in our work provide a good background for further investigations with distinct experimental models to extend the presented findings.

## 6. Conclusions

To conclude, we demonstrated that both G232E/R482L-*LMNA* mutations cause dysregulation in coordination with pathways that control cell cycle dynamics and muscle differentiation, induce mitochondrial uncoupling, and decrease glycolytic activity in differentiated myotubes. These alterations may contribute to mutation-induced muscle tissue pathology and should be considered as an important factor in the pathogenesis and progression of *LMNA*-related muscle diseases.

## Figures and Tables

**Table 1:** Primers for site-directed mutagenesis.

Primer Name	Primer Sequence (5′-3′)
h*LMNA*_R482L forward	TTACC(**G**/T)GTTCCCACCAAAGTTCACCCTGAAGG *
h*LMNA*_R482L reverse	GGAAC(**C**/A)GGTAAGTCAGCAAGGGATCATCTCCA *
h*LMNA*_G232E forward	CTGGTGGAGATTGACAATG(**G**/A)GAAGCAGCGTGAGTTTGAG *
h*LMNA*_G232E reverse	CTCAAACTCACGCTGCTTC(**C**/T)CATTGTCAATCTCCACCAG *

* Original nucleotide is printed in bold.

**Table 2:** Primers for sequencing analysis of mutagenesis.

Primer Name	Primer Sequence (5′-3′)
h*LMNA*_R482L seq-forward	GCTGGTCGAGTACCAGGAGCTTCTGGACATCA
h*LMNA*_R482L seq-reverse	GCCGTAGGCAGGCTGTTCCCGCAGCCCCAGGT
h*LMNA*_G232E seq-forward	GGATGAGATGCTGCGGCGG
h*LMNA*_G232E seq-reverse	GCTGGGCAGAGAGGCTGTCG

**Table 3:** Number of differentially expressed genes (DEGs) found in both—pathways and DEGs of control transgenic samples (*p* < 0.0001) ^1^.

	Number of Genes in Overlap		
	WT vs. G232E	WT vs. R482L	R482L vs. G232E
Myogenesis (Hallmark database)	29	15	14
Muscle structure development	54	16	23
Muscle system process	48	20	24
Muscle contraction	41	18	21
Striated muscle cell differentiation	38	11	18
Muscle cell differentiation	41	11	19
Muscle cell development	30	10	14
Myofibril assembly	20	8	11
Cellular component assembly involved in morphogenesis	20	8	11
Striated muscle contraction	20	10	10
Anatomical structure formation involved in morphogenesis	39	14	20
Muscle filament sliding	13	8	7
Actomyosin structure organization	20	8	11
Actin filament based process	31	ns	15
Sarcomere organization	13	ns	7
Myotube differentiation	16	ns	9
Muscle fiber development	14	ns	9
Actin mediated cell contraction	ns	8	ns
Regulation of muscle contraction	ns	7	ns

^1^ Pathway analysis was performed on upregulated DEGs in WT/G232E, WT/R482L, and R482L/G232E pairs and combined into one table that represent upregulated pathways. GO biological processes and hallmark databases were used for pathway analysis with false discovery rate 0.05.

## Supplementary Materials

The following are available online at https://www.mdpi.com/2073-4425/11/9/1057/s1, Figure S1. Principal component analysis (PCA) plots of 24 samples; Figure S2. Heat map of normalized read counts for 24 samples and 14,321 genes; Figure S3. Relative mtDNA content in C2C12 transgene lines analyzed by real-time PCR amplification. Figure S4. Glycolytic capacity of myotubes; Figure S5: The fraction of h*LMNA*-positive cells in differentiated cultures. Table S1. Primers used for mtDNA quantification; Table S2. Full list of differential-expressed genes. Table S3. Full list of pathways that were significantly different between transgene lines; Table S4. Sequences of primers.
