# Supplementary material for: LMNA Mutations G232E and R482L Cause Dysregulation of Skeletal Muscle Differentiation, Bioenergetics, and Metabolic Gene Expression Profile
Source: Genes (Basel). 2020 Sep 7;11(9):1057. doi: 10.3390/genes11091057 (PMC7563596; doi:10.3390/genes11091057)
Supplement: Supplementary file 1 [file genes-11-01057-s001.pdf]

Supplementary figures.

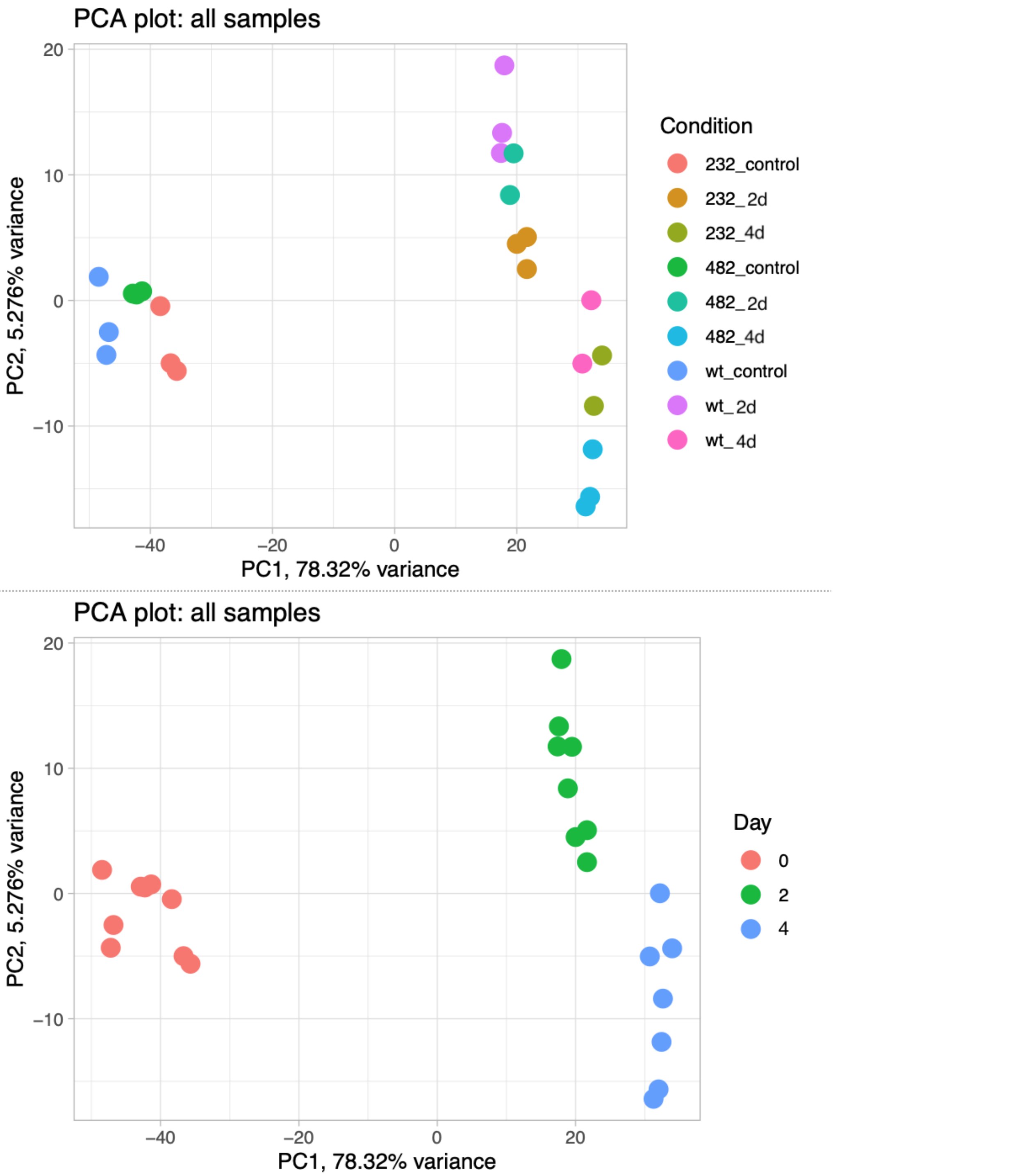

Suppl. Figure S1. Principal component analysis (PCA) plots of 24 samples. “Control” – undifferentiated myoblasts, “2d” – day 2 of differentiation, “4d” – day 4 of differentiation, terminal stage. For each biological condition there are 2 or 3 replicates. PCA made on regularized log (rlog) transformed counts. Samples on PCA plot on the top of figure are colored according to its condition; whereas samples on PCA plot at the bottom are colored with respect to the day of differentiation. Both PCA plots showed primarily clustering of samples into 3 big groups depending on day of muscle formation.

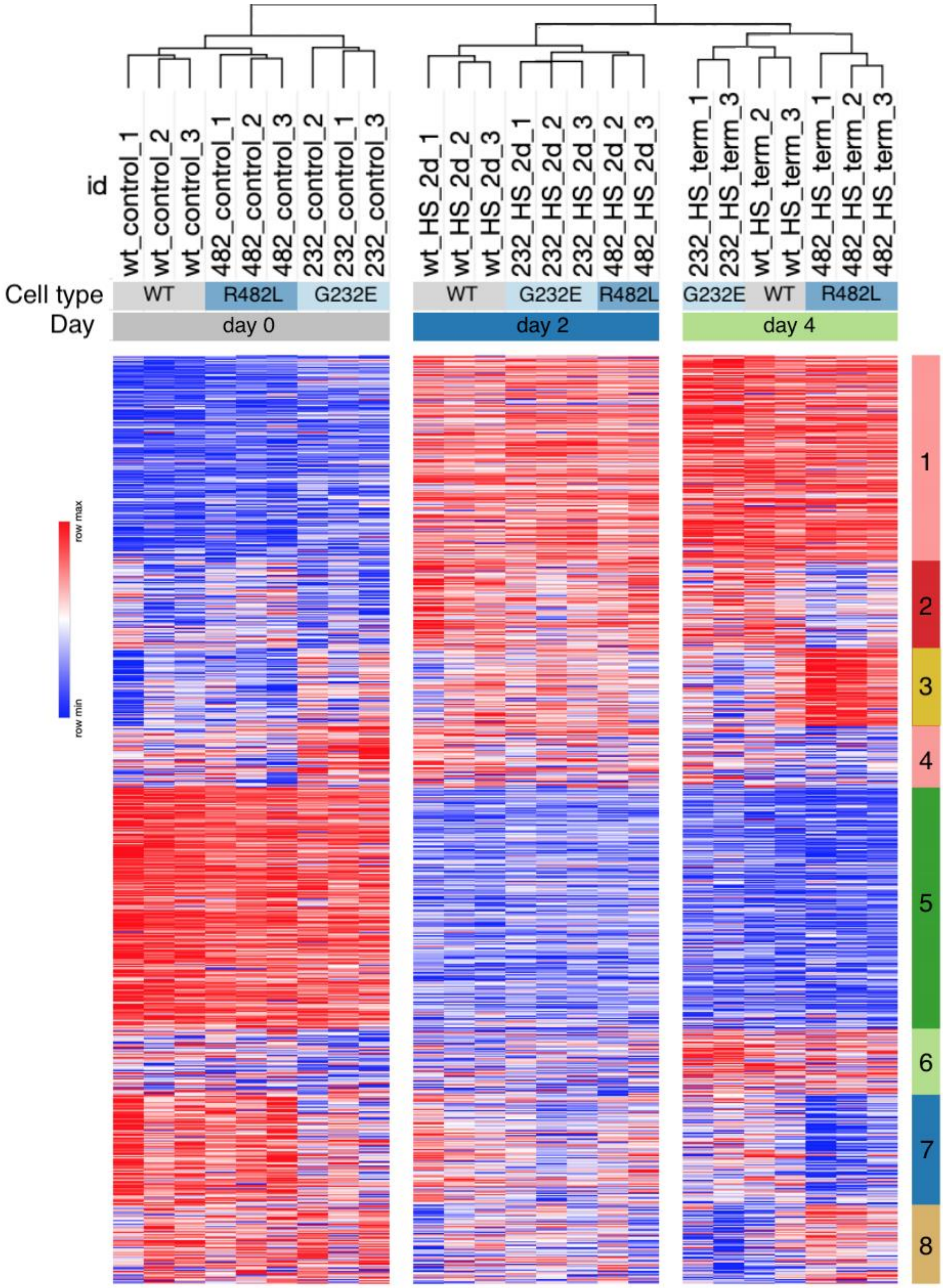

Suppl. Figure S2. Heatmap of normalized read counts for 24 samples and 14,321 genes. Raw counts were log-transformed and quantile normalized followed by filtering of low counts. Each biological condition (9 ones) represented by 2 or 3 biological replicates after outlier exclusion. Hierarchical clustering was applied on samples (top of figure S2) and showed the separation of samples explained mostly by days of differentiation; however, difference was observed between mutant transgenic cell lines also. Genes were clustered in 8 groups based on their expression similarity using k-means method: roughly 50% of genes are found in clusters 1 and 5 and responsible for the processes of myoblasts differentiation and cell cycle termination respectively.

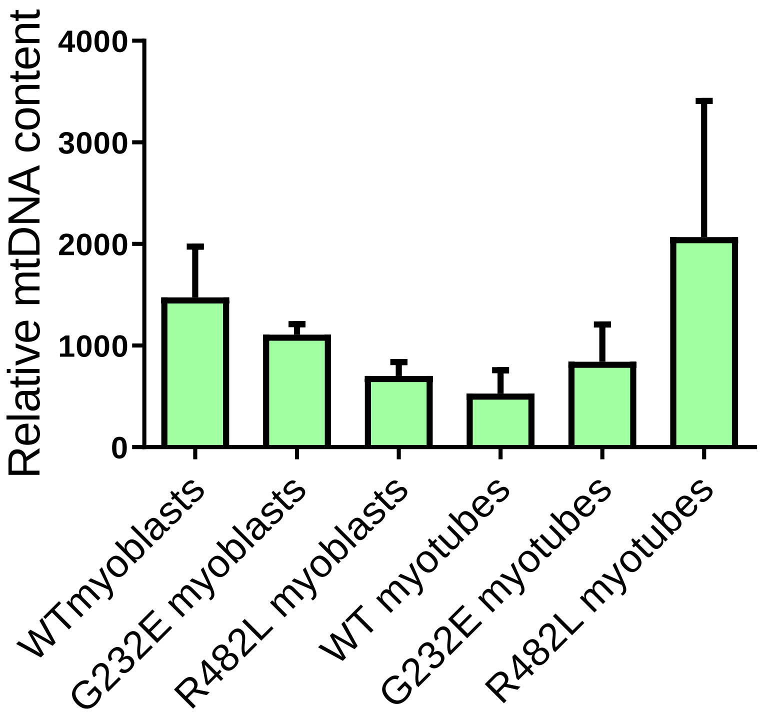

Suppl. Figure S3. Relative mtDNA content in C2C12 transgene lines analyzed by real-time PCR amplification. The relative mtDNA content is quantified from total DNA as the copy number of mtDNA, normalized to the copy number of single nuclear gene Kcnj13. The data are presented as mean with SEM. Significance was determined by ANOVA (Tukey's multiple comparisons) using GraphPad Prism version 8.4.1. No significant differences were found between groups.

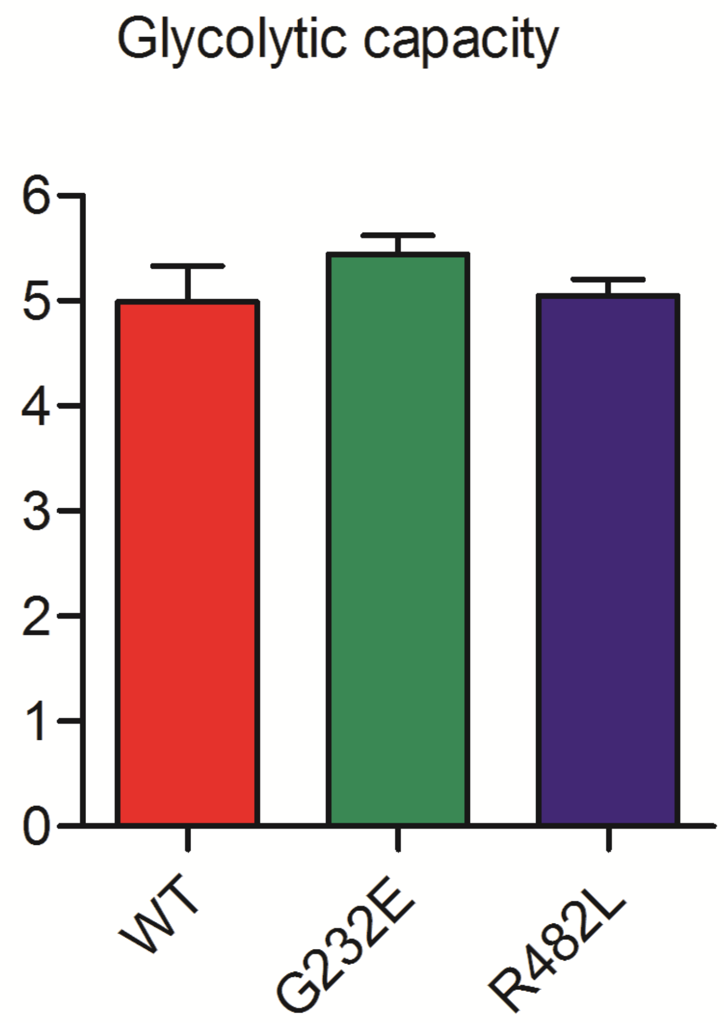

Suppl. Figure S4 Glycolytic capasity of myotubes

Glycolytic capacity was calculated as the basal ECAR subtracted from the highest ECAR used to meet cellular demands after ATP synthase is inhibited

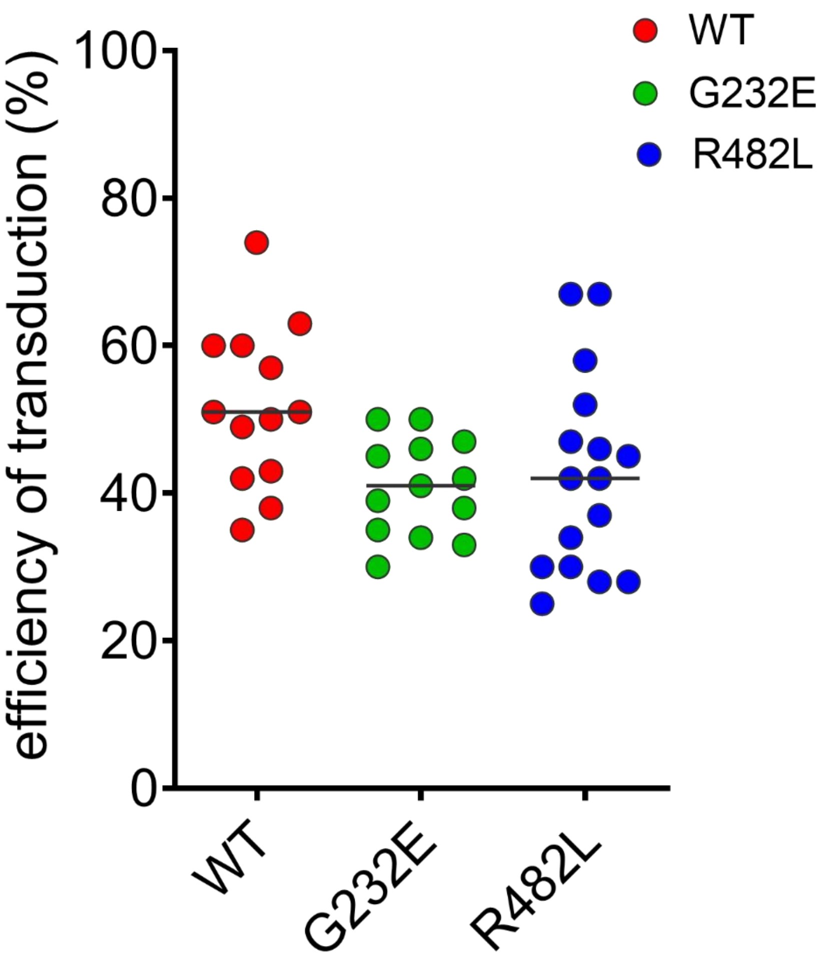

Suppl. Figure S5. The fraction of hLMNA-positive cells in differentiated cultures. Cells were stained with anti lamin A antibody recognizing only human lamin A. Nuclei were visualized by DAPI staining. The representative numbers of pictures (indicated in figure legend) were taken for each tested condition.

The efficiency of transduction is calculated as a percent of LMNA-positive cells in culture.

Target DNA	Primer Name	Primer Sequence (5'- 3')
**The murine mitochondrial fragment**	musMT553F23 forward	GCCAGAGAACTACTAGCCATAGC
	musMT668R23 reverse	AGCAAGAGATGGTGAGGTAGAGC
**Kcnj13**	mus4987F25 forward	GGATGAGAGAGAGAAGCACAAGTGG
	mus5140R25 reverse	CTGTATGACCAACCTTGGACATGAT

Suppl, Table S1 Primers used for mtDNA quantification

Mouse gene	Orientation	Sequence 5'-3'
GAPDH	Fw	GGATCTGACGTGCCGCCTG
	Rv	GAAGGTGGAAGAGTGGGAGTTGC
Mymk	Fw	CCTGTGATGGGCCTGGTTTGTC
	Rv	GGTTCATCAAAGTCGGCCAGTGC
Mymx	Fw	GGACCACTCCCAGAGGAAGGA
	Rv	GGACCGACGCCTGGACTAAC
Myog	Fw	GAGACATCCCCCTATTTCTACCA
	Rv	GCTCAGTCCGCTCATAGCC
Myh1	Fw	GCTGAGAGAAGCTACCACATT
	Rv	ACAAAGGCGTAGTCGTATGG
Myh3	Fw	CTATGCCACCTTCGCTACAACAG
	Rv	CAGTGCAGAGACGGTTTGGAAGG
Myh7	Fw	TGCCCGATGACAAAGAAGAG
	Rv	GTCACCGTCTTGCCATTCT

Suppl, Table 4 Primers for RT-qPCR
